# Advancing past ketamine: emerging glutamatergic compounds for the treatment of depression

**DOI:** 10.1007/s00406-024-01875-z

**Published:** 2024-08-29

**Authors:** Florian Freudenberg, Christine Reif-Leonhard, Andreas Reif

**Affiliations:** 1 Department of Psychiatry, Psychosomatic Medicine and Psychotherapy, University Hospital, Goethe University Frankfurt, Heinrich-Hoffmann-Str. 10, 60528 Frankfurt am Main, Germany; 2 Fraunhofer Institute for Translational Medicine and Pharmacology ITMP, Theodor-Stern-Kai 7, 60596 Frankfurt Am Main, Germany

**Keywords:** Depression, MDD, Rapid acting antidepressant, Glutamate, NMDA, AMPA

## Abstract

Changes in glutamatergic neuroplasticity has been proposed as one of the core mechanisms underlying the pathophysiology of depression. In consequence components of the glutamatergic synapse have been explored as potential targets for antidepressant treatment. The rapid antidepressant effect of the NMDA receptor antagonist ketamine and subsequent approval of its S-enantiomer (i.e. esketamine), have set the precedent for investigation into other glutamatergic rapid acting antidepressants (RAADs). In this review, we discuss the potential of the different glutamatergic targets for antidepressant treatment. We describe important clinical outcomes of several key molecules targeting components of the glutamatergic synapse and their applicability as RAADs. Specifically, here we focus on substances beyond (es)ketamine, for which meaningful data from clinical trials are available, including arketamine, esmethadone, nitrous oxide and other glutamate receptor modulators. Molecules only successful in preclinical settings and case reports/series are only marginally discussed. With this review, we aim underscore the critical role of glutamatergic modulation in advancing antidepressant therapy, thereby possibly enhancing clinical outcomes but also to reducing the burden of depression through faster therapeutic effects.

## Introduction

The first evidence for an involvement of glutamatergic neuroplasticity in depression emerged in preclinical studies of the late 1980s and early 1990s, which demonstrated that hippocampal long-term potentiation is diminished by inescapable stress [1] and that NMDA inhibitors exert effects comparable to those of traditional antidepressants [2]. Since then, the glutamatergic neuroplasticity hypothesis of depression has been supported by countless preclinical and clinical studies. As a comprehensive summary is out of the scope of this article (the avid reader is referred to more focused reviews on this topic, see e.g. [3–14]), here we will only review the most relevant findings.

A number of magnetic resonance spectroscopy studies, investigating Glx (glutamate + glutamine) levels have been performed in patients with depression. A meta-analysis of these studies identified a moderate reduction in Glx levels in the medial prefrontal cortex of medicated (but not unmedicated) patients with depression [15]. Moreover, altered expression levels of glutamate receptor genes and proteins has been described in animal models (e.g., following acute or chronic stress, with and without treatment with antidepressants), and depression-like behavioural changes have been observed in various glutamate receptor mouse mutants (reviewed e.g. in [9, 10, 16]). Altered expression of glutamate receptors has also been described in postmortem brain samples of patients with depression (see e.g. [17–20]) and a more recent study showed that glutamate receptor gene expression was particularly affected in women with depression [21]. Genome-wide association studies have linked various glutamate receptor genes (e.g., *GRIK5*, *GRM5*, *GRM8*) to depression and, more intriguingly, these studies have found associations with pathways related to synaptic plasticity [22]. This accumulated evidence led to the (glutamatergic) neuroplasticity hypothesis of depression, which has been elaborated in a number of review articles (e.g. [3, 23, 24]) and has since been further refined.

In the year 2000 Berman et al. reported rapid antidepressant effects of a single sub-anaesthetic dose of the NMDA receptor antagonist ketamine [25]. A follow-up trial by Zarate et al. 2006 confirmed the rapid antidepressant effect of ketamine and showed sustained antidepressant potential for at least one week even in patients that did not respond to traditional antidepressants [26]. The antidepressant potential of ketamine has been repeatedly confirmed and its mechanism of action (MoA) that goes beyond simple NMDA receptor antagonisms has been and remains being explored (reviewed e.g. in [12, 27, 28]). Since the discovery of the antidepressant efficacy of ketamine, the involvement of the glutamatergic system and glutamatergic neuroplasticity in depression has become more or less indisputable [12]; indeed, glutamatergic neuroplasticity as a mechanistic basis for depression seems more plausible and possibly superior to the monoaminergic hypothesis.

**Fig. 1:**
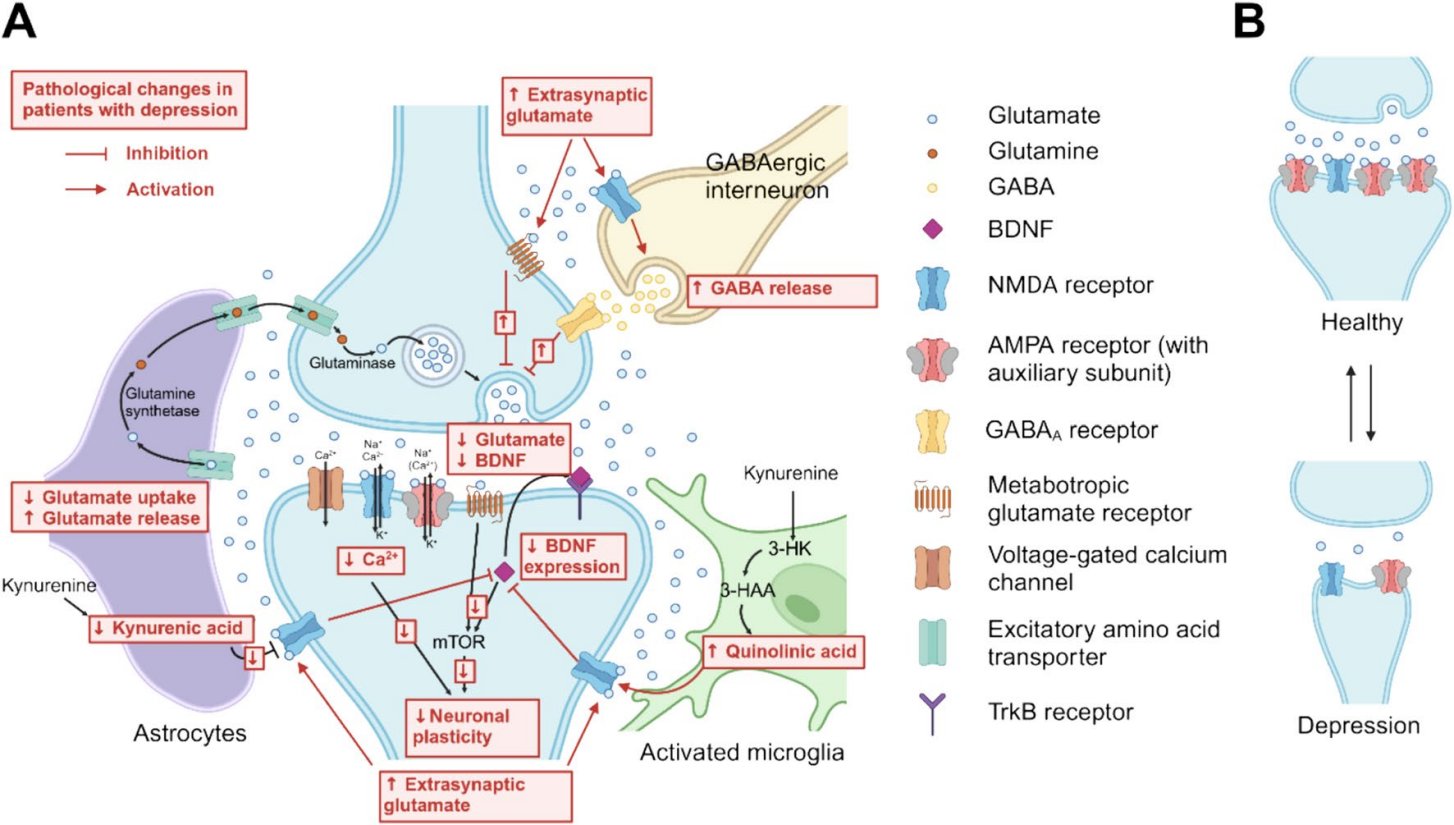
Changes in glutamatergic signalling in patients with depression. **A** Schematic of a glutamatergic synapse (blue) including key signalling mechanisms and molecules. Pathological changes described in depression are shown in red. The accumulation of extrasynaptic glutamate (due to reduced glutamate uptake and increased glutamate release in astrocytes) leads to inhibition of presynaptic glutamate release (through increased activation of presynaptic metabotropic glutamate receptors and GABA-A receptors), and thus reduced activation of postsynaptic AMPA and NMDA receptors, which in turn leads to reduced activation of downstream signalling pathways (including via BDNF-mTOR and calcium-activated signalling pathways), ultimately reducing neuronal plasticity. Inhibition of BDNF on the postsynaptic side also occurs through increased activation of extrasynaptic NMDA receptors (due to increased extrasynaptic glutamate and increased synthesis of quinolinic acid in activated microglia). **B** The described reduction in neuroplasticity lead to a long-term reduction in postsynaptic AMPA receptors and thus decreased synaptic efficacy, resulting in long-term structural reduction of the synapse. For various substances affecting the glutamatergic system, antidepressant efficacy has been demonstrated by mitigating, preventing, or reversing various shown pathological changes. For example, ketamine primarily inhibits extracellular NMDA receptors, thus preventing the GABAergic-mediated reduction in glutamate release and inhibition of BDNF expression. Additionally, hydroxynorketamine, a metabolite of ketamine, enhances synaptic activity by activating AMPA receptors. AMPAkines exert their effects through increased AMPA receptor activity, while inhibitors of metabotropic glutamate receptors aim to prevent the inhibition of presynaptic glutamate release. The figure was created with Biorender.com

At the core of the neuroplasticity hypothesis is the idea that various pathological stimuli, such as stress or inflammation, trigger signalling cascades that lead to pathological changes in glutamatergic signalling, thereby weakening the neuronal plasticity of glutamatergic synapses. Specifically, under pathological conditions, there is reduced uptake and increased release of glutamate by astrocytes. This leads to extrasynaptic spillover of glutamate, which causes increased activation of metabotropic glutamate receptors and disinhibition of GABAergic interneurons (through activation of NMDA receptors on these interneurons) at the presynaptic site. This results in reduced presynaptic glutamate release, leading to reduced activation of synaptic NMDA and AMPA receptors, which leads to a reduced activation of a number of downstream signalling pathways, including protective signalling cascades e.g. via BDNF. At the postsynaptic site, glutamate spillover stimulates extrasynaptic NMDA receptors, which inhibit the mTOR pathway thereby also reducing BDNF-mediated neuroprotection. Stimulation of extrasynaptic NMDA receptors are also the main mechanism underlying the glutamatergic involvement of neuroinflammation in depression, by increased release of quinolinic acid from activated microglia. The described changes in consequence lead to reduced synaptic plasticity, precipitated in the reduction of synaptic AMPA receptors further weakening glutamatergic signalling, resulting in a long-term structural reduction in the size of glutamatergic synapses (Fig. 1) (also see one of the many focused reviews on this topic, e.g. [4–14, 23, 24]). While there is a high degree of consensus favouring changes in glutamatergic neuroplasticity as a basis for depression, the neuroplasticity hypothesis still remains at the level of a hypothetical model pending indisputable experimental proof.

### From monaminergic drugs to rapid acting antidepressants

Following the introduction of tricyclic antidepressants in the 1950s and 1960s, the development of substances with a novel MoA essentially stagnated for decades. While tetracyclic antidepressants, MAO inhibitors, as well as serotonin, norepinephrine, and dopamine reuptake inhibitors followed, all these molecules share a MoA primarily based on the modulation of monoaminergic signal transduction. Specifically, the MoA of these substances is mainly based on increasing the concentration of serotonin, but also norepinephrine or dopamine in the synaptic cleft [29, 30]. Of note, a substantial proportion of patients (up to about 55% in the most stringent assessments" [31]) show treatment resistance. However, the term treatment-resistant depression (TRD) is fundamentally misleading; according to the common EMA/FDA definition TRD means "inadequate response to two sequential antidepressants of different classes at adequate doses and duration" [31]. In effect, this means "resistant to traditional antidepressant therapy targeting the monoaminergic system." Also common to these substances is their latency of several weeks to exert an antidepressant effect, although the increase in monoaminergic neurotransmission occurs more or less immediately. The duration of this latency of effect has been assessed and defined differently over the decades: while originally a latency of up to six weeks was assumed, later meta-analyses suggested a latency of about two weeks until the onset of antidepressant effects (see e.g. [32, 33]). The molecular basis for this delayed effect is not fully clear, but a multitude of molecular/cellular changes have been described and include transcriptomic, proteomic, and epigenetic changes as well as activation of transcription factors and signalling cascades that promote different molecular functions including Cytochrome P450 activity, energy metabolism, lipid metabolism, synaptic plasticity and neurotransmitter systems, activation of neurotrophins (particularly BDNF), immune/inflammatory processes, and many more (see e.g. [3, 34–36] for further details).

Regardless of whether the latency is one, two, or even six weeks, the long period until clinically relevant improvement is achieved, especially when considering the dosage adjustment phase, remains unsatisfactory from a clinical perspective, and might become distressing for the patient. Therefore, the development of novel substances with an innovative MoA and rapid onset is one of the most important goals in psychopharmacotherapy. With the approval of intranasal esketamine (trade name "Spravato") in 2019 by both the FDA and EMA, an antidepressant with a novel MoA and rapid onset was introduced to the market for the first time. Other substances with similar or fundamentally different MoA, meeting the criterion of a rapid-acting antidepressant (RAAD) are under active development.

## Rapid acting antidepressants (RAADs)

RAADs are characterized by a rapid onset of antidepressant effects, i.e., within hours to days and by high efficacy after one (or a few) applications [37]. Moreover, RAADs show marked efficacy against "treatment-resistant" depression (TRD [31]) or difficult-to-treat depression (D2TD [38]), though it is unclear if this is a genuine effect, as most studies (at least for ketamine) were in fact exclusively performed in TRD/D2TD patients (i.e. there is no baseline for the effect in non-TRD patients). However, each of these intuitively understandable features remains vaguely defined. For example, it is unclear whether there is truly a rapid and sustained antidepressant effect ("rapidly effective") or if the true antidepressant efficacy occurs later, with the presence of rapid but transient mood-enhancing effects ("rapid-acting"). [31]

It remains unresolved whether RAADs are more effective than other antidepressants, as there are only few head-to-head comparisons between RAADs and conventional antidepressants. Moreover, the interaction of RAADs with co-administered traditional antidepressants is not always clear, though for ketamine synergistic effects have been described (e.g. [39, 40]). Finally, considering newer long-term studies [41], at least for ketamine, it must also be questioned whether one or two doses of RAAD are truly sufficient, especially since the antidepressant effect seems to consolidate only after several doses [42]. Due to the heterogeneity of RAADs, these points are likely to be assessed differently across different substance classes.

### Substance classes of RAADs

In their review article published in 2018 [43], Witkin et al. proposed the following RAAD classes according to their MoA: (i) NMDA receptor antagonists, (ii) metabotropic glutamate receptor (mGluR) 2/3 antagonists, (iii) scopolamine, (iv) negative allosteric modulators (NAM) of α5 subunit-containing GABA-A receptors, and (v) psychedelics. Five years later, this list should be expanded to include positive allosteric modulators (PAM) of AMPA receptors (AMPAkines), GABA PAMs, partial agonists of the µ-opioid receptor (mOR-pA), and kappa-opioid receptor antagonists (kOR-A).

In a database search conducted in June 2024 at clinicaltrials.gov for currently active, recruiting, and not yet recruiting phase II and III studies on depression (i.e. “Depressive Disorder” or “Unipolar Depression” or synonyms), the following presently investigated substances with RAAD potential were identified: esketamine (Spravato®), NMDAE (an NMDA enhancer; not further defined), BI 1569912 and NBI-1070770 (negative allosteric modulators of GluN2B), esmethadone, ketamine, nitrous oxide (N_2_O), Apimostinel, D-cycloserine, NRX101 (a fixed combination of D-cycloserine/lurasidone) (all NMDA receptor modulators); TS-161 (mGluR2/3 antagonist); aticaprant, navacaprant (kOR-A); buprenorphine (mOR-pA); morphine (μ-opioid receptor agonist); propofol, HS-10353, Allopregnanolone (GABA-PAM); N,N-dimethyltryptamine, LSD, psilocybin, and derivatives (psychedelics, i.e. predominantly acting as 5-HT_2A_ agonists). While substances acting on a range of pharmacological targets are tested for their potential as RAADs, here we only focus on RAADs acting on the glutamate system; these include the substances identified in the database search, but also those that have been investigated in the last years, but are not actively studied at the current time (e.g. Arketamine, AXS-05 [Auvelity®], onfasprodil).

## RAADs targeting the NMDA receptor

Without a doubt, the majority of RAADs investigated at the moment target the glutamate system. Most likely, this can be attributed to the fact that the RAAD potential of ketamine has already been postulated more than 30 years ago and that the S-enantiomer of ketamine (i.e. esketamine) is approved as an intranasal formulation for the treatment of TRD, thus being the first approved RAAD.

As the potential of (es)ketamine to act as a RAAD has been repeatedly documented and review (reviewed e.g. in [12, 27, 28]) and given that esketamine is an already approved antidepressant, we will focus here on glutamatergic drugs beyond ketamine. Yet, we would like to highlight some important aspects regarding the antidepressant efficacy of ketamine that will become important when discussing these other drugs.

Ketamine, as well as esketamine, act as non-competitive antagonists of the NMDA receptor on inhibitory, GABAergic interneurons in the hippocampus. This inhibition leads to a reduced release of GABA thereby leading to reduced inhibition of the excitatory neuron. In consequence, there is an increased release of glutamate, leading to activation of postsynaptic AMPA receptors. This, along with direct NMDA inhibition, results in the inhibition of intracellular signalling cascades (CaMKIII deactivation) leading to increased BDNF release [27]. A hallmark study showed that ketamine rapidly activates mTOR, thereby leading to the formation of new synapses [44]. Both effects result in increased neuronal plasticity, which counteracts the reduced, stress-related neuroplasticity (see above) in depression [45]. However, mere NMDA blockade alone does not sufficiently explain the antidepressant effect of (es)ketamine, as other NMDA antagonists like memantine do not show a comparable effect [46]. In fact, it has been shown that some of these NMDA receptor inhibition-independent effects might be caused by ketamine metabolites acting as AMPA receptor activators [47]. Of note, also differences in pharmacokinetics and affinity to different NMDA receptor subpopulations have been discussed to explain the distinct clinical effects of ketamine and memantine [48].

One of the main side effects of ketamine and esketamine is dissociation. It is still unclear whether dissociation and the antidepressant effect are interlinked – being part of the same MoA – or if they are distinct phenomena. A common belief is that dissociation correlates with the antidepressant effect of (es)ketamine. However, data from three independent studies contradict this assertion [49–51]. In this context, it is important to distinguish between short-term euphoric and long-term antidepressant effects. While the intensity of dissociation may correlate with a better antidepressant response after one day [49] (although one study found that very strong dissociation [CADSS > 15] was associated with worse response [50]), no significant correlation was found between dissociation and the antidepressant effect of (es)ketamine after day 3, day 7, or day 28. This suggests that the acute antidepressant effect (up to 24 h after infusion) of (es)ketamine could be due to an acute "trip" (which could correspond to acute NMDA antagonization). However, the long-term antidepressant effect does not correlate with dissociation and might rather be due to neuroplastic, AMPA, or BDNF-induced phenomena. As a result, the search for other substances acting on the glutamate system that demonstrate the antidepressant efficacy of (es)ketamine without the side effect of dissociation is in full swing.

### Arketamine

In addition to esketamine, the R-enantiomer of ketamine (i.e. arketamine), has been investigated as a potential antidepressant based on preclinical studies [52]. This was accompanied by high hopes for at least equally good antidepressant efficacy, with fewer side effects, especially with regard to dissociation. A pilot open-label phase II study on 7 TRD patients showed a rapidly occurring and significant antidepressant effect [53]. However, another small, double-blind crossover study by the same research group [54] showed no significant difference from placebo. At the beginning of 2023, *atai Life Sciences* announced that a phase IIa study (ClinicalTrials.gov ID NCT05414422), which compared two dosages of arketamine against placebo in TRD patients, was negative with respect to the primary endpoint (statistically significant MADRS reduction 24 h after infusion). Currently, it is unclear whether the development of arketamine as an antidepressant will continue.

### Hydroxynorketamine

In a seminal report, Zanos et al. [47] showed that hydroxynorketamine (HNK) the metabolite of ketamine is essential for the antidepressant activity of ketamine. They could further show that delivery of HNK in mice was sufficient to exert antidepressant-like effects, not by inhibition of NMDA receptors, but by activation of AMPA receptors [47]. The antidepressant-like effects in preclinical models has been repeatedly confirmed (see e.g. [55–57]). However, two independent clinical trials, one in patients with suicidal depression [58] and one in TRD patients [59], showed an inverse relationship of HNK levels post ketamine infusion with clinical outcome (i.e. lower HNK levels correlated with increased symptom improvement). In fact, clinical trials in relation to depression have not progressed beyond phase I (ClinicalTrials.gov ID NCT04711005), though HNK is currently in a phase II trial for neuropathic pain (ClinicalTrials.gov ID NCT05864053). Thus, it is unclear whether HNK will be further pursued for its antidepressant potential.

### AXS-05 (Auvelity®; bupropion/dextromethorphan fixed combination)

Dextromethorphan (DXM) is sold over-the-counter as a cough suppressant and does not cross the blood–brain barrier at typical dosages for this use. However, at sufficiently high blood levels (e.g., from misuse), DXM can be detected in the cerebrospinal fluid. In the brain DXM acts as an NMDA receptor antagonist, a sigma-1 receptor agonist, and a monoamine reuptake inhibitor [60]. To achieve sufficient levels, DXM is combined with an inhibitor of CYP2D6, the DXM metabolizing enzyme, such as quinidine or bupropion. While several studies on DXM in bipolar depression were negative [60] – likely due to the lack of combination with a CYP2D6 inhibitor – a small open-label study with TRD patients was positive [61]. This led to a clinical trial program by *Axsome Therapeutics* with a fixed combination of 45 mg DXM and 105 mg bupropion (AXS-05). Two controlled studies with AXS-05, ASCEND (against bupropion alone [62]) and GEMINI (against placebo [63]), were positive. In GEMINI, there was a significant separation of drug vs placebo regarding MADRS difference and remission after one or two weeks respectively. In the AXS-05 treated group of patients, 40% reached remission after six weeks (MADRS < 10). Whether the onset of action after one week justifies the label "rapidly effective" is more of a theoretical question; however, compared to conventional antidepressants, this timeframe is considerably shorter. The side effect profile of AXS-05 was generally comparable to that of other NMDA antagonists, but with generally fewer adverse effects, particularly regarding dissociation. Based on these two studies, AXS-05 was approved by the FDA in 2022 and is available in the USA under the trade name "Auvelity." If and when Auvelity® will be available in Europe is currently unclear; given the EMA requirements on fixed combination products [64] and the described differences between the FDA and EMA for initial therapy approval for fixed combination products [65], the approval for Auvelity® in Europe might be a long shot. Other variations of DXM combinations, such as deuterated DXM (AVP-786) or a combination with quinidine (Nuedexta), are also in clinical development [66].

### Esmethadon (REL-1017)

Esmethadone (also: Dextromethadone; as investigational drug by *Relmada Therapeutics*: REL-1017) is the S-enantiomer of methadone and, unlike methadone, has only a very low and probably irrelevant affinity for opioid receptors. Instead, esmethadone acts as an NMDA receptor antagonist, blocking the MK-801 binding site of the receptor with relatively high affinity. As with other RAADs, the effect of esmethadone in preclinical models seems to depend on mTOR and to be mediated by BDNF (reviewed in [67]). Increased circulating BDNF levels following esmethadone treatment were also found in phase I study (i.e. performed in healthy subjects) [68]. Surprisingly few preliminary data have been published on esmethadone, even though *Relmada Therapeutics* has launched an extensive study program (Reliance-II and -OLS; Relight). The first clinical trial, a placebo-controlled phase II study in patients with at least moderate depression, was published in 2022 [69]. In addition to good tolerability, a superiority of both tested esmethadone dosages (25 or 50 mg/day orally) in terms of reduction of MADRS was shown by the fourth day, with comparatively high effect sizes (d = 0.8 and 0.9, respectively). The effect lasted for a week after the last dose was administered, over a dosing period of one week. The data from the currently ongoing phase III trials (ClinicalTrials.gov IDs NCT06011577 and NCT04855747) are eagerly awaited.

### Nitrous oxide (N_2_O, laughing gas)

As its colloquial name “laughing gas” suggests, nitrous oxide has an acute (albeit short-term) mood-enhancing (euphoric) effect even in healthy individuals. Therefore, nitrous oxide has been increasingly misused, especially among adolescents and young adults [70]. Nitrous oxide is a non-competitive antagonist at the NMDA receptor but also acts on other molecular targets, particularly by exerting opioidergic effects and by inhibition of AMPA and kainate receptors [71, 72].

An initial blinded, placebo-controlled crossover trial with 20 TRD patients found a significant effect of a 50%/50% nitrous oxide/oxygen mixture on the Hamilton Depression Scale (HAMD) after two and 24 h compared to placebo [73]. The HAMD items that showed the best response were depressed mood, guilt, psychic anxiety, and, interestingly, suicidal ideation. Three patients fully remitted (7 or more points reduction in the HDRS-21 score) after nitrous oxide treatment. In a later phase II study the same research group was able to confirm the efficacy of nitrous oxide and further found that 25% nitrous oxide also worked comparably well but with fewer side effects [74]. This study also revealed that the effects of nitrous oxide treatment (both 50% and 25%) lasted for at least two weeks. In another randomized control trial in TRD patients from China, the two week-long efficacy could not be confirmed; however, this study also found nitrous oxide to be effective after two and 24 h [75]. In a Canadian randomized controlled trial on bipolar disorder patients with current TRD a single-treatment with 25% nitrous oxide was only superior in the MADRS response rate in comparison to the control group (intravenous midazolam) after 2 h; another 2 h later (i.e. 4 h after treatment) and beyond, both treatment arms showed comparable response [76]. Yet, a small meta-analysis including this and the previous three studies (i.e. [73–76]) showed significant benefit of nitrous oxide treatment at 24 h post-treatment, but no significant effect after one week of treatment [72].

A Brazilian study examined the effect of repeated (twice weekly, over four weeks) nitrous oxide inhalations and found remarkable improvement in non-TRD patients [77]. A recently published systematic review also concluded a potential benefit of nitrous oxide treatment in depression and identified ten more ongoing studies for different indications (3 for depression, 3 for TRD, and 2 for bipolar disorder, one for PTSD and one for OCD) [78].

Since the studies published so far include relatively few patients, evaluating nitrous oxide as a RAAD is still premature. However, it is a well-controllable substance with a manageable side effect profile, and further studies are certainly justified and are ongoing (ClinicalTrials.gov IDs NCT05357040 and NCT03869736) or planned (ClinicalTrials.gov IDs NCT06382389 and NCT05710887).

### D-cycloserine

D-cycloserine acts as a partial agonist at the co-agonistic glycine-binding site of the NMDA receptor. However, at high doses, D-cycloserine acts as an NMDA antagonist. D-cycloserine has been investigated as a cognitive enhancer in schizophrenia or as an enhancer of psychotherapy effects [79], and a preliminary study showed positive effects in TRD [80]. NRX-101, a fixed-dose combination of D-cycloserine with the second-generation antipsychotic lurasidone, is being studied for efficacy in bipolar depression and suicidal ideation in mood disorders. At least one of these studies uses a sequential protocol with an initial ketamine infusion followed by oral NRX-101 therapy for continuation of treatment. If this strategy proves successful as supported by the results from this first trial [81], this would significantly facilitate efforts of both patients and practitioners. Results from another trial (ClinicalTrials.gov ID NCT03395392) are not yet published and an additional trial (NCT03396068) is ongoing.

### Other molecules targeting the NMDA receptor

Numerous other molecules that target the NMDA receptor have been investigated as potential antidepressants in animal models or in Phase I/II human studies (overview in [66]). However, very few have demonstrated a convincing, rapid, and sustained antidepressant effect. Potentially this is because these substances interact differently with the NMDA receptor in terms of site and mechanism; it is also possible that the non-competitive NMDA antagonism of (es)ketamine (which, unlike other substances, can only bind to the receptor in its open state) is just one aspect of a complicated MoA involving multiple molecular targets. Among the substances that also inhibit the NMDA receptor and were effective in pilot studies but then failed in confirmatory studies are the low-trapping NMDA receptor antagonist lanicemine (AZD6765) [82]. The GluN2B subunit-selective antagonist traxoprodil (CP-101,606) was also successful in a pilot study [83] but further development was stopped due to QTc prolongation. Other GluN2B antagonists like EVT-101 or rislenemdaz (MK-0657, CERC-301) are currently not being developed further due to regulatory holds or negative data respectively [66, 84]. The well-tolerated [85], negative allosteric GluN2B modulator onfasprodil (MIJ-821) was tested by Novartis as a RAAD for TRD and depression with suicidal ideation. While a Phase II study in TRD was positive (ClinicalTrials.gov ID NCT03756129), the study in patients with suicidal ideation (NCT04722666) was prematurely terminated without providing further information. Another Phase II trial is completed (NCT05454410), but no results have been published yet.

4-Chlorokynurenine (AV-101) a small molecule prodrug of 7-chlorokynurenic acid, which acts as a full antagonist at the glycine-binding site of the NMDA receptor. This drug joins the long list of substances that were effective in preclinical models but not in clinical studies. After a failed Phase II study [86], it is unlikely to be pursued further – at least for the indication of depression.

Rapastinel (development name: GLYX-13) binds as a NMDA receptor PAM at a site other than the NMDA or glycine-binding site and exhibits complex pharmacology. Unlike the other mentioned substances, rapastinel enhances glutamate-dependent activation of the NMDA receptor in the medial prefrontal cortex [87] and may also increase synaptic plasticity. A pilot study showed a rapid antidepressant effect of rapastinel [88] in patients who had not responded to another antidepressant. However, three Phase III studies conducted by *Allergan* (summarized in [89]) were negative. Thus, rapastinel is not being developed further, though other substances with a similar MoA, like apimostinel (GATE-202, NRX-1074) or zelquistinel (GATE-251, AGN-241751) continue to be investigated.

## Metabotropic glutamate receptor antagonists

In addition to NMDA receptors, other types of glutamate receptors have been suggested as potential RAAD targets, including the metabotropic glutamate receptors (mGluRs). The eight mGluRs (mGluR1-8) are G-protein-coupled glutamate receptors [90]. mGluR2 and 3 belong to Class II of the mGluRs and inhibit adenylate cyclase. They reduce NMDA activity and protect against glutamatergic excitotoxicity; their inhibition has led to very rapid, ketamine-like antidepressant effects in preclinical studies [91]. The mGlu2/3 antagonist TS-161 is safe and sufficiently bioavailable [92] and is currently under clinical investigation (ClinicalTrials.gov ID NCT04821271). However, a larger study with the mGlu2/3-NAM Decoglurant was negative [93], which has been attributed to factors other than the MoA.

Other mGluRs (e.g. mGluR5) have also shown promising potential in preclinical studies without any positive clinical studies (reviewed in [94, 95]).

## AMPAkines (AMPA-PAMs)

The MoA of (es)ketamine, as well as mGlu2/3 antagonists, converges on the activation of AMPA receptors [27, 91, 96], leading to increased BDNF release, TrkB activation, and enhanced neuronal plasticity. The involvement of AMPA receptors in depression is well documented and was extensively discussed in a previous review article by us [9]. Hence, the hypothesis that direct activation of AMPA receptors using AMPAkines could have an antidepressant effect is plausible [9, 97]. However, despite the solid preclinical evidence, only few clinical studies with AMPAkines have been performed. A small randomized, double-blind, placebo-controlled trial in patients with depression using the AMPAkine Org 26,576 did not reveal significant benefit of this drug compared to placebo [98]. One currently investigated compound is NBI-1065845/TAK-653 (ClinicalTrials.gov ID NCT05203341) [96], which has shown target engagement in initial studies and exhibits the properties of a psychostimulant [99]. Another AMPAkine, tulrampator (S-47445, CX-1632), was tested in a relatively large phase II study for efficacy in treatment-resistant depression (TRD) (NCT02805439); the results were negative and are unpublished but available online (https://clinicaltrials.servier.com/wp-content/uploads/CL2-47445-014-synopsis-report.pdf).

Whether the strategy of directly stimulating AMPA receptors or the BDNF pathway will prove worthwhile remains to be seen and requires further and larger clinical study programs. In this context, it also makes sense to consider the pharmacological modulation of AMPA receptor auxiliary subunits. These auxiliary subunits (including the TARP, Cornichon, and CKAMP proteins) interact directly with AMPA receptors and modulate several of their properties including receptor trafficking and receptor kinetics [100]. Disturbed AMPA receptor trafficking is also a key mechanism underlying glutamatergic mechanisms of depression and is also relevant in the context of the antidepressant efficacy of ketamine [101]. Therefore, it is not surprising that at least in preclinical models, the efficacy of ketamine has been linked to the AMPA auxiliary subunit TARP-γ8 and its interaction with PSD-95 [102]. However, though various negative and positive modulators of AMPA receptor auxiliary subunits have been identified and developed, specific studies on a potential antidepressant activity have yet to be conducted.

## Other potential RAADs interacting with the glutamatergic system

Beyond the above-described substances other substances that more indirectly interact with the glutamatergic systems have been classified as potential RAADs. The most promising of these substances that are the subject of a number of completed and still ongoing clinical trials are psilocybin (and other 5-HT2A partial agonists, i.e. psychedelics) [103–105], and the muscarinic cholinergic receptor antagonist scopolamine [106, 107]. Similarly to ketamine, the rapid antidepressant activity of both psilocybin and scopolamine has been assigned to changes in neural plasticity, involving increased glutamate release leading to elevated mTOR and BDNF activity and other downstream mechanism that promote synaptic plasticity [14, 107–110].

## Summary and conclusions

Ketamine and esketamine are well implemented glutamatergic RAADs in clinical practice and esketamine has been approved for use in TRD or for emergency treatment. In the USA, the dextromethorphan/bupropion combination is already on the market; another drug with NMDA receptor antagonistic effects and rapid antidepressant onset. Esmethadone is the next substance with potential for market introduction. Whether nitrous oxide, given its problematic administration and potential for abuse, will ever receive FDA or EMA approval remains to be seen. All other substances that target the glutamatergic system and have been studied in clinical trials have not led to convincing results both in term of onset of action and efficacy. Perhaps this is because modulation of isolated glutamatergic targets might not suffice to achieve antidepressant efficacy. Possibly other molecular effects, such as those present in (es)ketamine, must also be involved. Therefore, whether it will be possible to develop glutamatergic substances with a lower side effect profile (especially in terms of dissociation) and/or better efficacy than (es)ketamine remains an exciting question.
